# The Global Phylogeography of Lyssaviruses - Challenging the 'Out of Africa' Hypothesis

**DOI:** 10.1371/journal.pntd.0005266

**Published:** 2016-12-30

**Authors:** David T. S. Hayman, Anthony R. Fooks, Denise A. Marston, Juan C. Garcia-R

**Affiliations:** 1 Molecular Epidemiology and Public Health Laboratory, Hopkirk Research Institute, Massey University, Palmerston North, New Zealand; 2 Wildlife Zoonoses and Vector-borne Diseases Research Group, Animal and Plant Health Agency (APHA), Weybridge-London, United Kingdom; 3 Department of Clinical Infection, Microbiology & Immunology, Institute of Infection and Global Health, University of Liverpool, Liverpool, United Kingdom; Aix Marseille University, Institute of Research for Development, and EHESP School of Public Health, FRANCE

## Abstract

Rabies virus kills tens of thousands of people globally each year, especially in resource-limited countries. Yet, there are genetically- and antigenically-related lyssaviruses, all capable of causing the disease rabies, circulating globally among bats without causing conspicuous disease outbreaks. The species richness and greater genetic diversity of African lyssaviruses, along with the lack of antibody cross-reactivity among them, has led to the hypothesis that Africa is the origin of lyssaviruses. This hypothesis was tested using a probabilistic phylogeographical approach. The nucleoprotein gene sequences from 153 representatives of 16 lyssavirus species, collected between 1956 and 2015, were used to develop a phylogenetic tree which incorporated relevant geographic and temporal data relating to the viruses. In addition, complete genome sequences from all 16 (putative) species were analysed. The most probable ancestral distribution for the internal nodes was inferred using three different approaches and was confirmed by analysis of complete genomes. These results support a Palearctic origin for lyssaviruses (posterior probability = 0.85), challenging the ‘out of Africa’ hypothesis, and suggest three independent transmission events to the Afrotropical region, representing the three phylogroups that form the three major lyssavirus clades.

## Author Summary

Rabies virus kills tens of thousands of people globally each year and causes indescribable misery and family disturbance, especially in developing countries. Yet in much of the world there are related viruses, called lyssaviruses, which circulate among bats without causing conspicuous outbreaks. The greater diversity of African lyssaviruses has led to the hypothesis that Africa is the origin of these viruses. To test this hypothesis, the genetic data from 153 representative viruses from 16 available lyssavirus species from across the world dated between 1956 and 2015 were analysed. Statistical models were used to reconstruct the historical processes that lead to the contemporary distribution of these viruses. Our results support a Palearctic origin for lyssaviruses, not Afrotropic, and suggest three independent transmission events to Africa from the Palearctic region.

## Introduction

Determining the evolutionary history of viruses is fundamental to our understanding of the patterns and processes occurring during viral emergence and spread. Emergence and spread of viral diseases is a permanent threat in animal and public health and special attention has been given to fast-evolving RNA viruses due to the high mortality rates recorded worldwide. The family *Rhabdoviridae* contains a diverse variety of RNA viruses that replicate in vertebrates, invertebrates and plants. The vast majority of rhabdoviruses have invertebrate vectors that play a role in the transmission to plants, fishes or mammals. The lyssaviruses, which cause the disease rabies, are unique within these negative-sense, single-stranded RNA viruses because they do not require arthropod vectors and are well-adapted to their mammalian hosts [1].

The prototypic virus within the genus *Lyssavirus* is rabies virus (RABV) [2]. RABV has a global distribution (with the exception of Australasia, Antarctica, and some islands). The principal reservoir of RABV is the domestic dog (*Canis familiaris*), although RABV is enzootic in a number of wildlife Carnivora, including fox, mongoose, raccoon, and skunk populations. RABV variants undergo genetic adaptation to each particular host, resulting in new clades or biotypes relating to the local fauna [3–11]. Well-established wildlife Carnivora reservoirs for RABV are apparently absent in South America and Australasia. However, lyssaviruses are present in both of these regions and throughout the rest of the world in bat hosts (Chiroptera).

All lyssaviruses have been isolated from bats, with the exception of Mokola virus (MOKV) and Ikoma lyssavirus (IKOV), and phylogenetic analyses suggest all lyssaviruses have bat origins [12–16]. RABV has only been identified in bats in the Americas and is the only lyssavirus detected circulating in American bat species. This observation is in direct contrast to the rest of the world, where RABV has not been detected in bat populations. The greatest diversity of lyssaviruses occurs in bats in Eurasia and on the African continent. The species are divergent enough that sera raised against specific virus species often do not neutralize other virus species and are divided into phylogroups (PG) [14, 17, 18].

Rabies virus is a member of phylogroup 1 (PG1). European bat lyssavirus-1 (EBLV-1) and 2 (EBLV-2), Bokeloh bat lyssavirus (BBLV), Irkut (IRKV), Aravan (ARAV) and Khujand (KHUV) viruses all belong to PG1. These PG1 viruses have all been isolated from bats in Eurasia, as has West Caucasian Bat Virus (WCBV) from PG3. Lleida bat lyssavirus (LLEBV) is a tentative new species yet to be recognised by the International Committee on Taxonomy of Viruses (ICTV). LLEBV has been identified from a bat in Spain and is most closely related to the African Ikoma lyssavirus (IKOV) genetically in PG3 [19–21]. Gannoruwa bat lyssavirus (GBLV), also awaiting ICTV recognition as a new species, has recently been isolated from fruit bats in Sri Lanka, and phylogenetic analysis indicated that it is most closely related to RABV (PG1) [22]. In Australasia the only lyssavirus identified is Australian bat lyssavirus (ABLV), also a PG1 virus, which circulates within the Australian bat populations. In Africa, however, all three phylogroups are represented: Duvenhage virus (DUVV) from PG1, Lagos bat virus (LBV), MOKV and Shimoni bat virus (SHIBV) from PG2 [13], and IKOV [23] from PG3.

The observation that the greatest genetic diversity of lyssaviruses is in Africa has led to the hypothesis that Africa is the continent where lyssaviruses originated, most likely from an African bat reservoir [24]. This hypothesis is founded on sound observations, though does not address the lack of known reservoir(s) for MOKV, and was proposed despite a lack of understanding of the ecology of MOKV and many other lyssavirus species. Here, the hypothesis that lyssaviruses had their origins in Africa was tested by using a probabilistic phylogeographical approach, which provides additional insights into the historical biogeography of lyssaviruses.

## Methods

We used 153 nucleoprotein (N) gene sequences from the 14 recognized and 2 putative lyssavirus species (LLEBV, GBLV) in these analyses (final dataset, see below), along with complete genome sequences from all 16 (putative) species when appropriate for confirmatory analyses. Lyssavirus sequences were mostly derived from bats, with exception of MOKV and IKOV (see Introduction). Using bat-derived sequences was particularly important for RABV because the evolutionary history was less likely to be confounded by the global spread of RABV by human movement of terrestrial carnivores and the post-war RABV epidemics in wildlife [25–27]. Sequences represent serially sampled data; the earliest sequence from 1956 and the last from 2015, including the most recently available sequences from GBLV and LLEBV, spanning a 59-year period.

The dataset was aligned in ClustalX2.1 [28] and inspected by eye. Bayesian Markov Chain Monte Carlo (MCMC) implemented in BEAST software v.1.8.3 [29] was used for phylogenetic analysis and estimation of divergence times. A codon partition strategy was implemented with a general time reversible (GTR) model of substitution with gamma distributed variation in rates amongst sites and a proportion of sites assumed to be invariant according to the Akaike criterion in Modeltest [30]. The lower number of substitutions per site in EBLV-1 (S1 Fig) [31] compared across the tree can potentially cause problems for the estimation of the posterior probabilities and other parameters. To mitigate this issue, the number of EBLV-1 sequences was reduced in the final dataset (S1 Table). Divergence times were estimated using a strict clock model in BEAST assuming an underlying coalescent process with a constant population size. To be more conservative in our estimates of the divergence times and assuming that purifying selection has removed deleterious mutations from rate estimates in short timeframes [32–34], a 2.3 x 10^−4^ substitution rate estimated by Bourhy and colleagues [35] was used. An uncorrelated lognormal relaxed clock was also considered because it assumes that the substitution rate along branches is not correlated.

Parameter effective sample sizes were visualized in Tracer v.1.5 (http://tree.bio.ed.ac.uk/software/tracer/). The results from two independent runs with 5x10^7^ MCMC length of chain were combined. The first 10% of maximum clade credibility (MCC) trees were discarded as burn-in in TreeAnnotator v1.8.3 [36]. Final trees were visualized in FigTree v.1.4.2 [37]. The fit of each analysis (strict and lognormal) were evaluated with Bayes Factors in Tracer v.1.5 (http://tree.bio.ed.ac.uk/software/tracer/).

**Fig 1:**
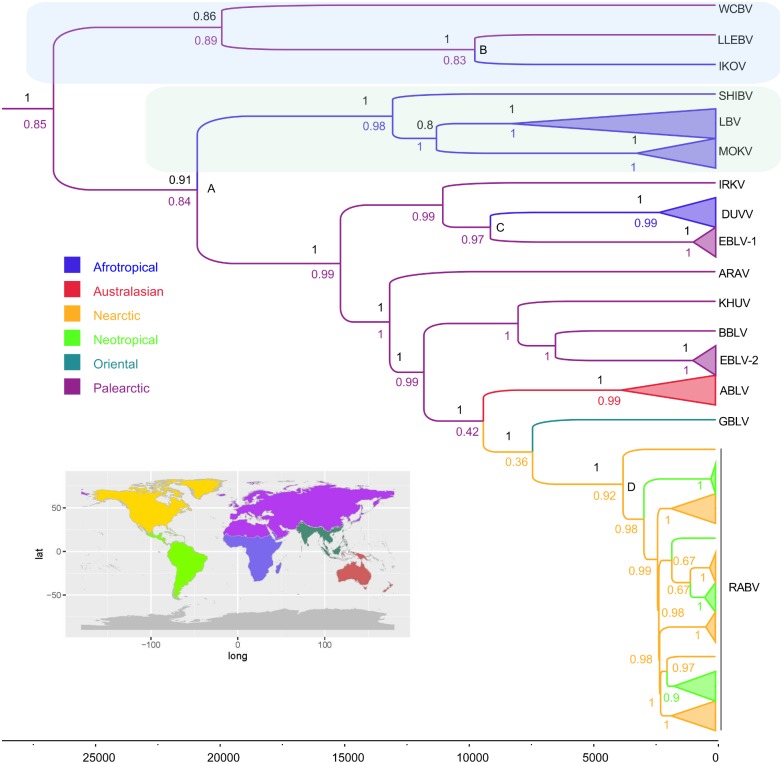
Evolutionary relationships between lyssaviruses. The time-scaled phylogeny was generated from 153 nucleoprotein gene sequences and inferred with a Lognormal relaxed-clock Bayesian analysis using BEAST. Branch colours correspond to ecozones shown on the inset map. Support values corresponding to Bayesian posterior probabilities (above branches) and states probabilities from the different assigned ecozones (below branches) are indicated for key nodes. The time scale in years is shown. Phylogroups 3 (green, top) and 2 (blue) are shaded and key nodes discussed in the text labelled A-D. Virus names are *Mokola virus* (MOKV); *Australian bat lyssavirus* (ABLV); *European bat lyssavirus-1* (EBLV-1); *European bat lyssavirus-2* (EBLV-2), *Irkut* (IRKV), *Aravan* (ARAV), *Khujand* (KHUV); *West Caucasian Bat Virus* (WCBV); *Lagos bat virus* (LBV); *Duvenhage virus* (DUVV); *Shimoni bat virus* (SHIBV); *Bokeloh bat lyssavirus* (BBLV); *Ikoma virus* (IKOV); *Lleida virus* (LLEBV); *Gannoruwa bat lyssavirus* (GBLV); *Rabies virus* (RABV).

Ancestral distributions were first performed in BEAST as part of the analysis for the estimations of the divergence times. The analysis assumed the forward and reverse rates to be symmetrical (Mk1). For comparison, our sampling was reduced to contain a single complete genome representative of each taxon, including GBLV and LLEBV, and we performed a RAxML analysis [38] to obtain a Maximum Likelihood phylogenetic tree. This phylogenetic tree was used to infer the most probable ancestral distribution for the internal nodes with Likelihood and Parsimony approaches and restriction of equal probability for all state changes with the Mk1 model using Mesquite v3.04 [39]. For ancestral state analyses viruses were categorized by the terrestrial ecozone from which they were isolated [35]. The major ecozones were Afrotropical, Palearctic, Oriental and Nearctic and Neotropic (both Americas) (Fig 1). Note that we use the term Oriental for the often named Indomalaya region but do not partition the Palearctic into Palearctic, Saharo-Arabian and Sino-Japanese regions [40] because no viruses were available from these latter two regions. Because RABV can be identified in more than one ecozone, its distribution category was treated as uncertain for the Likelihood analysis whilst polymorphic for the Parsimony analysis. Summary trees are presented in the main text and detailed trees with additional information, such as with all tip labels, are presented as figures in the supplementary information.

## Results

**Fig 2:**
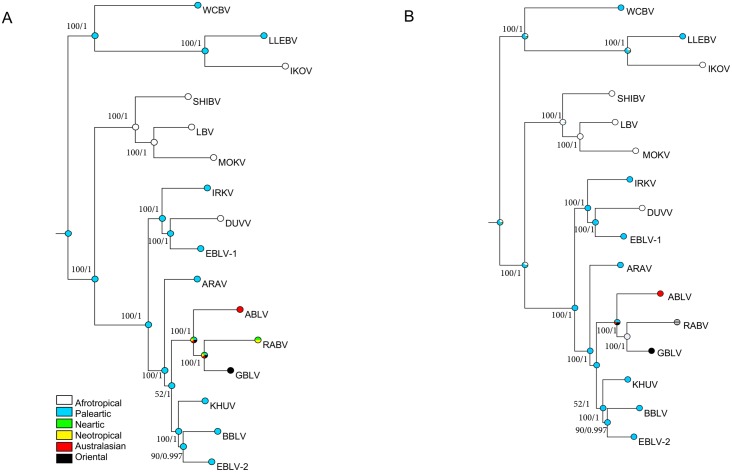
Ancestral state reconstruction using complete genomes of the 16 *Lyssavirus* species. The reconstruction was based on the Maximum Likelihood tree and using the parsimony (A) and likelihood (B) models in Mesquite v.3.0.4. Coloured pie-charts represent proportions generated from the different assigned states of the character (see colour legends). Grey terminal pie-chart in likelihood analysis indicated a polymorphic state that was coded as uncertain in the data matrix for RABV species. Support values are indicated above branches and correspond to bootstrap and posterior probabilities, respectively. Virus names are detailed in Fig 1.

Using a panel of lyssavirus N genes with global distributions there was strong support for the overall tree topology (Fig 1). The topology was confirmed through complete genome analysis (Fig 2). The uncorrelated relaxed clock (-25893.0, Fig 1) outperformed the strict clock model (-25999.2, S2 Fig). The results were supportive of a Palearctic origin for the lyssaviruses (posterior probability (PP) = 0.85, Fig 1, PP = 0.86 S2 Fig, strict clock). The results suggest there were three independent transmission events from the Palearctic to the African region, one each from the three putative phylogroups (Fig 1). One event led to the presence of IKOV in Africa (node B). Another event led to the distinct PG2 virus clade (SHIBV, LBV and MOKV) having their current African distribution (node A). It has been proposed that EBLV-1 had its origins in Africa, being closely related to DUVV, whereas our analysis suggested there was greater support for DUVV being a subsequent introduction into the Afrotropical region from the Palearctic (PP = 0.96, node C). Likelihood and Parsimony analyses of individual viral species genomes both provided support for these results (Fig 2). Our analysis supports an easterly spread of lyssaviruses to Australasia, the Oriental realm and the Americas. The inclusion of GBLV into our dataset supports previous findings that GBLV shares a most recent common ancestors (MRCA) with RABV (PP = 1), but support for this ancestral state node was the weakest (PP = 0.36). ABLV (previously the most closely related lyssavirus species to RABV) shares a MRCA with GBLV, although support for this ancestral state is also weak (PP = 0.42) (Fig 1).

Though not the aim of our analysis, we also estimate the time to the MRCA (tMRCA). The uncertainty around these estimates is large; however they suggest that these events probably took place tens of thousands of years ago. The three median tMRCA for the branching events relating to the Palearctic to Afrotropical region clades are 20820 years ago (95% highest posterior density, HPD, 3995 to 166820) for the PG1/PG2 ancestors (node A), 9676 year ago (1102 to 83408 95% HPD) for IKOV and LLEBV MRCA (node B) and 9048 years ago (1405 to 77181 95% HPD) for the DUVV and EBLV-1 MRCA (node C). The results also suggest that once RABV entered the Americas there was widespread dispersal of RABV between the Neotropical and Nearctic regions (Fig 1). Estimated tMRCA for the earliest RABV in our dataset is 3726 years ago (593 to 31478 95% HPD, node D).

## Discussion

Our analyses support a Palearctic origin for the lyssaviruses (PP = 0.85). This is despite the high diversity of lyssaviruses found in the Africotropical region. The support for most state probabilities is high, suggesting there is strong geographic and temporal structure to lyssavirus evolution as previously demonstrated by RABV in non-volant Carnivora species [41, 42]. Our estimation of the temporal origins of extant lyssaviruses varies depending on whether we used a fixed rate of evolution or estimated the rate using a relaxed clock. This does not, however, affect the phylogeographic inferences from this analysis because of the accurate estimation of evolutionary relationships among species (Figs 1 and 2). These analyses reject the hypothesis that lyssaviruses emerged as viruses of bats in Africa and suggest three distinct emergence events from the Palearctic region into the Afrotropical region (Fig 1).

There is topological support for the current phylogroups (PG1-3), each with monophyletic origins (Fig 1). A PG 3, with lower support, contains species IKOV, LLEBV and WCBV. PG 2 has a single Afrotropical ecozone range composed by SHIBV, MOKV and LBV. PG 1 divides into two major lineages, one which contains IRKV, EBLV-1 and DUVV, and another lineage that includes the Palearctic (ARAV, BBLV, KHUV and EBLV-2), Australian (ABLV), Oriental (GBLV) and American (RABV) lyssavirus species. A possible mechanism for the distribution of these viruses may be provided by considering the hosts of WCBV and LLEBV (PG3) and potential hosts of EBLV-1 and DUVV (PG1). Each of these viruses has been detected in the same bat genus, *Miniopterus*, thus these bats may have played a role in the inter-continental spread of lyssaviruses. This bat genus occurs in the Afrotropical and Palearctic ecozones, though species such as *Miniopterus schreibersi* previously thought to be distributed across both ecozones are now recognized to have more complex taxonomies [43]. However, the Palearctic species, *Miniopterus schreibersi*, from which WCBV was isolated in the Russian Caucasus and LLEBV and EBLV-1 were identified in Spain, occurs in both North Africa and Eurasia. WCBV is yet to be detected in Africa, but specific antibodies against WCBV have been detected in African *Miniopterus* in Kenya, providing possible evidence of a bat lyssavirus species circulating in both Africa and Europe [14, 44, 45]. There is no evidence of cross-neutralization between WCBV and IKOV (most closely related lyssavirus to WCBV), suggesting the antibodies detected in the *Miniopterus* spp in Kenya were WCBV specific [46]. The genetically close relationship between EBLV-1 and DUVV has been used as evidence of viruses from Africa entering Europe [47–49]. However, in our analysis, a greater support for transmission of EBLV-1 from the Palearctic to the Afrotropical zone was observed. The observation that both EBLV-1 and DUVV share a common ancestor with IRKV (Palearctic) strengthens this finding. The isolation of SHIBV from *Hipposideros vittatus* in Kenya [13] is the first lyssavirus isolation from a bat of the genus *Hipposideros*. *Hipposideros* has a broad distribution in the Old World from tropical Africa through to China, although like *Miniopterus* each species has a more specific distribution. Further sampling within this taxon will determine if SHIBV has crossed from the Palearctic to Afrotropical ecozones, as may be the case from the limited data available from WCBV [13] and our analyses (Fig 1).

Our results were confirmed through complete genome analyses with single representatives from each species. Despite this strong support for our conclusion that lyssaviruses have spread from the Palearctic region, it should be recognized that two of the most divergent viruses, WCBV and LLEBV, isolated from Russia [14] and Spain [20] respectively, may influence these findings [50, 51] and the ancestors of the *Lyssavirus* genus themselves may have originated from outside the Palearctic region, indeed even within the Afrotropical ecozone. The relationships of RNA viruses can be influenced by the different processes, such as through high evolutionary rates and subsequent purifying selection [52, 53]. We observed that EBLV-1 had reduced nucleotide substitutions per site compared to other viruses. The earliest EBLV-1 sequence is from 1968 and the sequences are taken from a relatively large geographic area (ranging from Ukraine to Spain), however EBLV-1 has a restricted host range. EBLV-1 has only ever been detected in the serotine bat (*Eptesicus serotinus*) outside Spain. In Spain, it has been reported from six European insectivorous bat species through active survey, although the majority were *E*. *serotinus* [47, 54]. Therefore, future studies should determine if host restriction is reducing the rate of nucleotide substitution and by what mechanism. The data from Spain may be evidence of this virus expanding into new niches and undergoing a bottleneck that reduced genetic diversity during the process. However, of the relatively well studied bat lyssaviruses, EBLV-2 has even greater known host restriction to *M*. *daubentonii*, and narrower geographic range, so alternative mechanisms may be responsible for the reduced substitution rate we estimated. As more full length genomes from more viruses become available, especially from bats, better inference can be made regarding evolutionary relationships for lyssaviruses [22]. In particular, we suggest that future studies aim to discover more viruses from Africa, Asia and the eastern Palearctic region.

Other significant dispersal events observed from this dataset are from the Palearctic to Australasia and between the Nearctic and Neotropical ecozones in both directions. These analyses provide support for the spread of lyssaviruses, in an easterly direction, from the Palearctic to Australasia and subsequent colonization into the Americas. Australia is free of RABV within its wildlife population with only occasional cases of imported human rabies [55–58], however, isolation of ABLV from sick bats [59], people [60–63], and through surveillance in bats [64–66] suggest ABLV is well established. Indeed, two distinct ABLV lineages apparently circulate, one now isolated from all four species of Australian Pteropodidae and another from an insectivorous bat species, *Saccolaimus flaviventris* [59, 64, 65, 67]. The isolation of GBLV from *Pteropus medius* in Sri Lanka in 2015 was significant because it is more closely related to RABV than any of the other Old World lyssaviruses currently identified. The host reservoir of GBLV (*P*. *medius*) is interesting for a number of reasons when considering spread of lyssaviruses between ecozones. Firstly, ABLV has been isolated from all four *Pteropus* spp in Australia. Secondly, members of the *Pteropus* genus are present throughout Asia and Australasia, providing a possible mechanism for transmission between the two ecozones. Thirdly, serosurveillance of bat populations in Asia has detected lyssavirus-specific antibodies, yet no virus had been isolated [16]. However, the ABLV host *S*. *flaviventris* has also been reported in Papua New Guinea [68] suggesting other pathways may exist.

Our analyses of the RABV data provide strong support for transmission between the American ecozones (Fig 1). This may be due to two, not mutually exclusive mechanisms. One is that despite recent evidence of host phylogeny constraining inter-species virus transmission with the USA [69], RABV has been isolated from over 23 bat species in the USA alone. Host signatures for species variants exist, but adaptation is not sufficient to prevent cross-species transmission [69]. Therefore, mutations in the RABV genome may have led to reduced host restriction and RABV being able to spread more easily between bats in both North and South American locations. An additional mechanism for this finding may be the hosts themselves. There are highly sociable, numerous, and/or migratory bats which occur throughout the Americas, such as *Tadarida brasiliensis*, *Lasiurus cinereus*, and *Eptesicus fuscus*. These migratory and widespread bat species of the Americas may have rapidly disseminated RABV between ecozones, enabling the promiscuous RABV to rapidly exploit unoccupied niches. Furthermore, the presence of *L*. *cinereus* in both Hawaii and British Columbia (http://www.iucnredlist.org/details/11345/0) demonstrates how a migratory species may be a potential vector of RABV or a RABV ancestor from the Palearctic to the Americas.

Our molecular clock analyses provide support for the hypothesis that RABV was circulating in the bat populations of the Americas before the arrival of Europeans in the late 15^th^ Century. Previously it has been claimed that Spanish conquistadors reported attacks by bats on humans and that native Americans knew that cauterisation may prevent disease development [70]. The median tMRCA for GBLV and RABV is >7,000 years ago. The median tMRCA for the first internal branch in RABV is 3726 years ago (593 to 31478 95% HPD). We are cautious when interpreting the ages of RNA viruses using molecular clock analysis because of the impacts of purifying selection on RNA viruses [52, 71], however purifying selection should push our dates further into the past rather than bring the estimates forward in time. Thus, we are confident that our analysis provides support for the probable presence of RABV in bats, and likely in the Americas, before the arrival of Europeans in 1492CE, because our lowest 95% HPD RABV tMRCA date is 1423CE and the median estimate is 3726 years ago in 1790 BCE.

The origins of RABV in dogs is debated and phylogenetic analyses have questioned the reports of RABV from ancient Greek references [70]. Our analyses demonstrate that lyssaviruses were almost certainly circulating in Palearctic bats at this time. Similarly, whether estimating evolutionary rates using relaxed clocks as in other analyses [35] or fixing the evolutionary rate, there appear to be extant RABV circulating in bats in the Americas at this time. Thus, it may be possible that the reports from 23^rd^ Century BC are due to an “extant” RABV if spillover had occurred from bats to terrestrial carnivores already. Future analyses of all extant lyssaviruses accounting for purifying selection may help elucidate these relationships further [52].

Lastly, extant Chiroptera bats likely originated in Asia/Europe and young clades are found in the Americas [72]. This biogeographic reconstruction reflects a similar pattern as the one found in the *Lyssavirus* genus. However, the time of divergence for extant Chiroptera is in Millions of years and there is no information to suggest co-speciation between bats and lyssavirus reflects a possible ancient origin of these viruses, as has been found in other groups (e.g. coronaviruses [71] and papillomaviruses [73]).

In conclusion, our analyses provide support for the monophyletic, Palearctic origins of lyssaviruses with dispersal from there to the rest of the world. And while three dispersal events have been from the Palearctic to the Afrotropical regions, arguably the dispersal events that led to the greatest impact on animal and human health are those eastward, where the RABV species appears to have evolved and dispersed globally from, leading to 23,000–93,000 human deaths a year [74]. Understanding why this lyssavirus, but not others, has emerged globally will provide insights into the processes that drive viral emergence.

## Supporting Information

S1 Table Partial nucleoprotein (N) or complete (C) lyssavirus genome sequences from GenBank used in the analysis. Virus names are as Fig 1.
(DOCX)

S1 Fig Phylogeny of a panel of lyssaviruses showing the slow rate of nucleotide substitutions for European bat lyssavirus-1 (green) compared to the other viruses (black). (PDF)

S2 Fig Evolutionary relationships between lyssaviruses based on the nucleoprotein (N) gene with a strict clock analysis using a fixed evolutionary rate of 2.3x10^-4^ (according to [35]) in BEAST. Support values corresponding to Bayesian posterior probabilities are indicated. Tips are labelled with the following information: Ecozone, follow by the_species name, country where it was isolated, GenBank accession number and year of isolation. Virus names and other details are as Fig 1.
(PDF)

S3 Fig Evolutionary relationships between lyssaviruses. The phylogenetic tree was generated from 153 nucleoprotein gene sequences and inferred with a Lognormal relaxed-clock Bayesian analysis using BEAST. Support values corresponding to Bayesian state probabilities from the different assigned ecozones are indicated. Tips are labelled with the following information: Ecozone, follow by the species name, country where it was isolated, GenBank accession number and year of isolation. Virus names and other details are as Fig 1.
(PDF)

S4 Fig Evolutionary relationships between lyssaviruses. The phylogenetic tree was generated from 153 nucleoprotein gene sequences and inferred with a Lognormal relaxed-clock Bayesian analysis using BEAST showing divergence times in years with 95% credible intervals. Branch colours correspond to ecozones shown on the map. The time scale is in years. Virus names are as Fig 1.
(PDF)

S5 Fig Ancestral state reconstruction using complete genomes of the 16 *Lyssavirus* species based on the Maximum Likelihood tree using likelihood models in Mesquite v.3.0.4. Coloured pie-charts represent proportions generated from the different assigned states of the character (see colour legends). The grey terminal pie-chart indicated a polymorphic state that was coded as uncertain in the data matrix for RABV species. Support values are indicated above branches and correspond to bootstrap and posterior probabilities, respectively. Virus names are as Fig 1.
(PDF)

S6 Fig Ancestral state reconstruction using complete genomes of the 16 *Lyssavirus* species based on the Maximum Likelihood tree and using the parsimony models in Mesquite v.3.0.4. Coloured pie-charts represent proportions generated from the different assigned states of the character (see colour legends). Support values are indicated above branches and correspond to bootstrap and posterior probabilities, respectively. Virus names are as Fig 1.
(PDF)
