# Supplementary material for: The Global Phylogeography of Lyssaviruses - Challenging the 'Out of Africa' Hypothesis
Source: PLoS Negl Trop Dis. 2016 Dec 30;10(12):e0005266. doi: 10.1371/journal.pntd.0005266 (PMC5231386; doi:10.1371/journal.pntd.0005266)
Supplement: S1 Table — Virus names are as Fig 1. (DOCX) [file pntd.0005266.s001.docx]

Table S1: Partial nucleoprotein (N) or complete (C) lyssavirus genome sequences from GenBank used in the analysis. Virus names are *Mokola virus* (MOKV); *Australian bat lyssavirus* (ABLV); *European bat lyssavirus-1* (EBLV-1); *European bat lyssavirus-2* (EBLV-2), *Irkut* (IRKV), *Aravan* (ARAV), *Khujand* (KHUV); *West Caucasian Bat Virus* (WCBV); *Lagos bat virus* (LBV); *Duvenhage virus* (DUVV); *Shimoni bat virus* (SHIBV); *Bokeloh bat lyssavirus* (BBL); *Ikoma virus* (IKOV); *Lleida* *virus* (LLEBV); *Gannoruwa bat lyssavirus* (GBLV); *Rabies virus* (RABV).

| Virus species | Geographical origin | Year | Genome | Species isolated from | GenBank reference | Reference |
| --- | --- | --- | --- | --- | --- | --- |
| ABLV | Australia: Queensland | 1996 | Complete | Human (ex. *Pteropus* sp.) | AF418014 | Warrilow et al. 2002 |
| ABLV | Australia: Queensland | 1996 | Complete | *Saccolaimus albiventris* | AF081020 | Gould et al. 2002 |
| ABLV | Australia: New South Wales | 1997 | Nucleoprotein to Polymerase | *Pteropus alecto* | AF006497 | Gould et al. 1998 |
| ARAV | Kyrghyzstan | 1991 | Complete | *Myotis blythi* | EF614259 | Kuzmin et al. 2003 |
| BBLV | Germany | 2009 | Nucleoprotein | *Myotis nattererii* | JF311903 | Freuling et al. 2011 |
| DUVV | South Africa | 1981 | Complete | *Miniopterus sp* | EU293120 | Delmas et al. 2008 |
| DUVV | South Africa | 1971 | Complete | Human (ex. bat) | EU293119 | Delmas et al. 2008 |
| DUVV | South Africa | 2006 | Complete | Human | EU623444 | Unpublished |
| DUVV | Kenya | 2007 | Nucleoprotein | Human | FJ515696 | van Theil et al. 2008 |
| DUVV | Zimbabwe | 1986 | Nucleoprotein | *Nycteris thebaica* | AY062080 | Johnson et al. 2002 |
| EBLV-1 | Denmark | 1987 | Nucleoprotein | *Eptesicus serotinus* | AY863374 | Davis et al. 2005 |
| EBLV-1 | Holland | 1998 | Nucleoprotein | *Eptesicus serotinus* | AY863367 | Davis et al. 2005 |
| EBLV-1 | Germany | 1982 | Nucleoprotein | *Eptesicus serotinus* | AY863351 | Davis et al. 2005 |
| EBLV-1 | Denmark | 1999 | Nucleoprotein | *Eptesicus serotinus* | AY863378 | Davis et al. 2005 |
| EBLV-1 | Germany | 1968 | Nucleoprotein | *Eptesicus serotinus* | EF157976 | Davis et al. 2005 |
| EBLV-1 | Germany | 1988 | Nucleoprotein | *Eptesicus serotinus* | AY863354 | Davis et al. 2005 |
| EBLV-1 | Germany | 1982 | Nucleoprotein | *Eptesicus serotinus* | AY863351 | Davis et al. 2005 |
| EBLV-1 | Spain | 2009 | Nucleoprotein | *Eptesicus serotinus* | HM212663 | Vazquez-Moron et al. 2011 |
| EBLV-1 | Spain | 2008 | Nucleoprotein | *Eptesicus serotinus* | HM212664 | Vazquez-Moron et al. 2011 |
| EBLV-1 | Germany | 1970 | Nucleoprotein | *Eptesicus serotinus* | AY863350 | Davis et al. 2005 |
| EBLV-1 | Slovakia | 2001 | Nucleoprotein | *Eptesicus serotinus* | AY863382 | Davis et al. 2005 |
| EBLV-1 | Holland | 1994 | Nucleoprotein | *Eptesicus serotinus* | AY863391 | Davis et al. 2005 |
| EBLV-1 | France | 2001 | Nucleoprotein | *Eptesicus serotinus* | AY863402 | Davis et al. 2005 |
| EBLV-1 | Holland | 1999 | Nucleoprotein | *Eptesicus serotinus* | AY863388 | Davis et al. 2005 |
| EBLV-1 | Holland | 1997 | Nucleoprotein | *Eptesicus serotinus* | AY863389 | Davis et al. 2005 |
| EBLV-1 | Holland | 1993 | Nucleoprotein | *Eptesicus serotinus* | AY863384 | Davis et al. 2005 |
| EBLV-1 | Holland | 1992 | Nucleoprotein | *Eptesicus serotinus* | AY863387 | Davis et al. 2005 |
| EBLV-1 | Holland | 1992 | Nucleoprotein | *Eptesicus serotinus* | AY863383 | Davis et al. 2005 |
| EBLV-1 | France | 2001 | Nucleoprotein | *Eptesicus serotinus* | AY863400 | Davis et al. 2005 |
| EBLV-1 | France | 2000 | Nucleoprotein | *Eptesicus serotinus* | AY863396 | Davis et al. 2005 |
| EBLV-1 | France | 1995 | Nucleoprotein | *Eptesicus serotinus* | AY863394 | Davis et al. 2005 |
| EBLV-1 | France | 2000 | Nucleoprotein | *Eptesicus serotinus* | AY863397 | Davis et al. 2005 |
| EBLV-1 | France | 1995 | Nucleoprotein | *Eptesicus serotinus* | AY863395 | Davis et al. 2005 |
| EBLV-1 | France | 2001 | Nucleoprotein | *Eptesicus serotinus* | AY863401 | Davis et al. 2005 |
| EBLV-1 | France | 2000 | Nucleoprotein | *Eptesicus serotinus* | AY863398 | Davis et al. 2005 |
| EBLV-1 | France | 1989 | Complete | *Eptesicus serotinus* | EU293112 | Delmas et al. 2008 |
| EBLV-1 | France | 2003 | Complete | *Eptesicus serotinus* | EU293109 | Delmas et al. 2008 |
| EBLV-1 | Germany | 1968 | Complete | *Eptesicus serotinus* | EF157976 | Marston et al. 2007 |
| EBLV-1 | France | 2004 | Nucleoprotein | *Eptesicus serotinus* | EU636794 | Dacheux et al |
| EBLV-1 | France | 2007 | Nucleoprotein | *Eptesicus serotinus* | EU636793 | Dacheux et al |
| EBLV-1 | France | 2006 | Nucleoprotein | *Eptesicus serotinus* | EU636792 | Dacheux et al |
| EBLV-1 | France | 2006 | Nucleoprotein | *Eptesicus serotinus* | EU636791 | Dacheux et al |
| EBLV-1 | France | 2007 | Complete | Cat | EU626552 | Dacheux et al |
| EBLV-1 | France | 2008 | Complete | *Eptesicus serotinus* | EU626551 | Dacheux et al |
| EBLV-2 | Finland | 2009 | Nucleoprotein | *Myotis daubentonii* | GU002399 | Jakava-Viljanen et al. 2010 |
| EBLV-2 | United Kingdom | 2002 | Nucleoprotein | *Myotis daubentonii* | AY212120 | Johnson et al. 2003 |
| EBLV-2 | United Kingdom | 2002 | Complete | Human (ex. bat) | EF157977 | Marston et al. 2007 |
| EBLV-2 | Holland | 1986 | Complete | *Myotis dasycneme* | EU293114 | Delmas et al. 2008 |
| EBLV-2 | Switzerland | 2002 | Nucleoprotein | NA | AY863408 | Davis et al. 2005 |
| EBLV-2 | Switzerland | 1993 | Nucleoprotein | *Myotis daubentonii* | AY863407 | Davis et al. 2005 |
| EBLV-2 | Holland | 1993 | Nucleoprotein | *Myotis dasycneme* | AY863404 | Davis et al. 2005 |
| EBLV-2 | Holland | 1989 | Nucleoprotein | *Myotis dasycneme* | AY863405 | Davis et al. 2005 |
| EBLV-2 | Finland | 1986 | Nucleoprotein | Human (ex. bat) | AY863406 | Davis et al. 2005 |
| GBLV | Sri Lanka | 2015 | Complete | *Pteropus medius* | KU244266 | Gunawardena et al. 2016 |
| IKOV | Tanzania | 2011 | Complete | Civet (*Civettictis civetta*) | NC_018629 | Marston et al. 2012 |
| IRKV | Russia: Baikal | 2002 | Complete | *Murina leucogaster* | EF614260 | Kuzmin et al. 2005 |
| KHUV | Tajikistan | 2001 | Complete | *Myotis mystacinus* | EF614261 | Kuzmin et al. 2003 |
| LBV | France (ex Togo or Egypt) | 1999 | Nucleoprotein | *Rousettus aegyptiacus* | EF547447 | Markotter et al. 2008 |
| LBV | Senegal | 1985 | Complete | *Eidolon helvum* | EU293108 | Delmas et al. 2008 |
| LBV | Kenya | 2007 | Complete | *Eidolon helvum* | EU259198 | Kuzmin et al 2008 |
| LBV | Kenya | 2009 | Complete | *Rousettus aegyptiacus* | GU170202 | Kuzmin et al 2010 |
| LBV | Nigeria | 1956 | Complete | *Eidolon helvum* | EU293110 | Delmas et al. 2008 |
| LBV | Central African Republic | 1974 | Nucleoprotein | *Micropteropus pussilus* | EF547449 | Markotter et al. 2008 |
| LBV | Zimbabwe | 1986 | Nucleoprotein | *Cat* | EF547450 | Markotter et al. 2008 |
| LBV | South Africa | 2004 | Nucleoprotein | *Epomorphorus wahlbergi* | EF547458 | Markotter et al. 2008 |
| LBV | South Africa | 2003 | Nucleoprotein | *Epomorphorus wahlbergi* | EF547451 | Markotter et al. 2008 |
| LBV | South Africa | 2004 | Nucleoprotein | *Mongoose* | EF547453 | Markotter et al. 2008 |
| LBV | South Africa | 1980 | Nucleoprotein | *Epomorphorus wahlbergi* | EF547454 | Markotter et al. 2008 |
| LBV | South Africa | 1982 | Nucleoprotein | *Epomorphorus wahlbergi* | EF547455 | Markotter et al. 2008 |
| LLEBV | Spain | 2012 | Complete | *Miniopterus schreibersii* | KY006983 | Marston et al. 2017 |
| MOKV | Zimbabwe | 1981 | Complete | Cat | Y09762 | Mercier et al 1997 |
| MOKV | Cameroon | 1974 | Complete | Shrew | EU293117 | Delmas et al. 2008 |
| MOKV | Central African Republic | 1983 | Complete | Rodent | EU293118 | Delmas et al. 2008 |
| MOKV | South Africa | 1997 | Nucleoprotein | Cat | AF074815 | Nel et al. 2000 |
| MOKV | South Africa | 1997 | Nucleoprotein | Cat | AF074816 | Nel et al. 2000 |
| MOKV | South Africa | 1998 | Nucleoprotein | Cat | AF074817 | Nel et al. 2000 |
| MOKV | South Africa | 2006 | Nucleoprotein | Cat | EF188811 | Sabeta et al. 2007 |
| MOKV | South Africa | 1996 | Nucleoprotein | Cat | AF074813 | Nel et al. 2000 |
| MOKV | South Africa | 1995 | Nucleoprotein | Cat | AF074814 | Nel et al. 2000 |
| MOKV | Zimbabwe | 1982 | Nucleoprotein | Cat | AF319514 | Nel et al. 2000 |
| MOKV | Zimbabwe | 1981 | Nucleoprotein | Dog | AF319515 | Nel et al. 2000 |
| MOKV | Zimbabwe | 1981 | Nucleoprotein | Dog | AF319516 | Nel et al. 2000 |
| MOKV | Zimbabwe | 1993 | Nucleoprotein | Cat | AF319517 | Nel et al. 2000 |
| MOKV | South Africa | 2005 | Nucleoprotein | Dog | EF188810 | Sabeta et al. 2007 |
| MOKV | South Africa | 1996 | Complete | Cat | AF074810 | Nel et al. 2000 |
| RABV | USA: Mississippi | 2004 | Nucleoprotein | *Tadarida brasiliensis* | GU644783 | Streicker et al 2010 |
| RABV | Argentina | 1997 | Complete | *Tadarida brasiliensis* | EU293116 | Delmas et al. 2008 |
| RABV | USA: Florida | 1987 | Nucleoprotein | *Lasiurus Intermedius* | AF351843 | Nadin-Davis et al., 2001 |
| RABV | USA: Connecticut | 1998 | Nucleoprotein | *Eptesicus fuscus* | AF351854 | Nadin-Davis et al., 2001 |
| RABV | USA: Connecticut | 1998 | Nucleoprotein | *Eptesicus Fuscus* | AF351860 | Nadin-Davis et al., 2001 |
| RABV | USA: Connecticut | 1998 | Nucleoprotein | *Lasiurus Borealis* | AF351857 | Nadin-Davis et al., 2001 |
| RABV | USA: Washington | 1987 | Nucleoprotein | *Eptesicus Fuscus* | AY039227 | Messenger et al., 2003 |
| RABV | USA: Pennsylvania | 1984 | Nucleoprotein | *Eptesicus Fuscus* | AY039226 | Messenger et al., 2003 |
| RABV | USA: Florida | 1988 | Nucleoprotein | *Myotis Austroriparius* | AY039225 | Messenger et al., 2003 |
| RABV | USA: Arkansas | 1991 | Nucleoprotein | *Lasiurus Borealis* | AY039224 | Messenger et al., 2003 |
| RABV | USA: California | 1986 | Nucleoprotein | *Myotis Californicus* | AF394873 | Messenger et al., 2003 |
| RABV | USA: Washington | 1995 | Nucleoprotein | *Myotis Californicus* | AF394872 | Messenger et al., 2003 |
| RABV | USA: California | 1987 | Nucleoprotein | *Myotis Californicus* | AF394871 | Messenger et al., 2003 |
| RABV | USA: Arizona | 1993 | Nucleoprotein | *Pipistrellus Hesperus* | AF394870 | Messenger et al., 2003 |
| RABV | USA: California | 1993 | Nucleoprotein | *Antrozous Pallidus* | AF394869 | Messenger et al., 2003 |
| RABV | USA: California | 1991 | Nucleoprotein | *Antrozous Pallidus* | AF394868 | Messenger et al., 2003 |
| RABV | USA: Pennsylvania | 1984 | Nucleoprotein | *Eptesicus Fuscus* | AY039229 | Messenger et al., 2003 |
| RABV | USA: Colorado | 1985 | Nucleoprotein | *Eptesicus Fuscus* | AY039228 | Messenger et al., 2003 |
| RABV | USA: Florida | 1988 | Nucleoprotein | *Lasiurus Intermedius* | AF394878 | Messenger et al., 2003 |
| RABV | USA: California | 1989 | Nucleoprotein | *Plecotus Townsendii* | AF394877 | Messenger et al., 2003 |
| RABV | USA: Florida | 1988 | Nucleoprotein | *Tadarida brasiliensis* | AF394876 | Messenger et al., 2003 |
| RABV | USA: California | 1990 | Nucleoprotein | *Myotis evotis* | AF394874 | Messenger et al., 2003 |
| RABV | USA: California | 1987 | Nucleoprotein | *Eptesicus Fuscus* | AF394887 | Messenger et al., 2003 |
| RABV | USA: Texas | 1986 | Nucleoprotein | *Lasiurus Borealis* | AF394886 | Messenger et al., 2003 |
| RABV | USA: Florida | 1988 | Nucleoprotein | *Lasiurus Borealis* | AF394885 | Messenger et al., 2003 |
| RABV | USA: Georgia | 1982 | Nucleoprotein | *Lasiurus Cinereus* | AF394884 | Messenger et al., 2003 |
| RABV | USA: Arkansas | 1991 | Nucleoprotein | *Pipistrellus Subflavus* | AF394881 | Smith et al, 2004 |
| RABV | USA: New York | 1984 | Nucleoprotein | *Lasionycteris Noctivagans* | AF394880 | Messenger et al., 2003 |
| RABV | Trinidad and Tobago | 1995 | Nucleoprotein | *Desmodus rotundus* | AF351852 | Nadin-Davis et al., 2001 |
| RABV | Mexico | 1991 | Nucleoprotein | *Tadarida brasiliensis* | AF352633 | Nadin-Davis et al., 2001 |
| RABV | Canada: British Columbia | 1992 | Nucleoprotein | *Myotis Lucifugus* | AF351834 | Nadin-Davis et al., 2001 |
| RABV | Canada: Saskatchewan | 1992 | Nucleoprotein | *Eptesicus fuscus* | AF351934 | Nadin-Davis et al., 2001 |
| RABV | Canada: British Columbia | 1972 | Nucleoprotein | *Eptesicus fuscus* | AF351833 | Nadin-Davis et al., 2001 |
| RABV | Canada: Saskatchewan | 1989 | Nucleoprotein | *Eptesicus fuscus* | AF351832 | Nadin-Davis et al., 2001 |
| RABV | Canada: British Columbia | 1988 | Nucleoprotein | *Eptesicus fuscus* | AF351831 | Nadin-Davis et al., 2001 |
| RABV | Canada: Ontario | 1993 | Nucleoprotein | *Eptesicus fuscus* | AF351830 | Nadin-Davis et al., 2001 |
| RABV | Canada: Ontario | 1993 | Nucleoprotein | *Eptesicus Fuscus* | AF351829 | Nadin-Davis et al., 2001 |
| RABV | Canada: Ontario | 1993 | Nucleoprotein | *Eptesicus Fuscus* | AF351828 | Nadin-Davis et al., 2001 |
| RABV | Canada: Ontario | 1993 | Nucleoprotein | *Lasiurus Cinereus* | AF351846 | Nadin-Davis et al., 2001 |
| RABV | Canada: Alberta | 1992 | Nucleoprotein | *Lasiurus Cinereus* | AF351845 | Nadin-Davis et al., 2001 |
| RABV | Canada: Ontario | 1994 | Nucleoprotein | *Lasiurus Borealis* | AF351844 | Nadin-Davis et al., 2001 |
| RABV | Canada: Manitoba | 1992 | Nucleoprotein | *Lasionycteris Noctivagans* | AF351842 | Nadin-Davis et al., 2001 |
| RABV | Canada: Ontario | 1980 | Nucleoprotein | *Lasionycteris Noctivagans* | AF351841 | Nadin-Davis et al., 2001 |
| RABV | Canada: Saskatchewan | 1988 | Nucleoprotein | *Lasionycteris Noctivagans* | AF351840 | Nadin-Davis et al., 2001 |
| RABV | Canada: British Columbia | 1992 | Nucleoprotein | *Myotis Lucifugus* | AF351839 | Nadin-Davis et al., 2001 |
| RABV | Canada: British Columbia | 1994 | Nucleoprotein | *Myotis Lucifugus* | AF351838 | Nadin-Davis et al., 2001 |
| RABV | Canada: British Columbia | 1979 | Nucleoprotein | *Lasionycteris Noctivagans* | AF351837 | Nadin-Davis et al., 2001 |
| RABV | Canada: British Columbia | 1992 | Nucleoprotein | *Myotis Californicus* | AF351836 | Nadin-Davis et al., 2001 |
| RABV | Canada: British Columbia | 1992 | Nucleoprotein | *Myotis evotis* | AF351835 | Nadin-Davis et al., 2001 |
| RABV | Brazil: Sao Paulo State | 2002 | Nucleoprotein | *Molossus Rufus* | AB201817 | Kobayashi et al., 2005 |
| RABV | Brazil: Sao Paulo State | 2000 | Nucleoprotein | *Molossus Abrasus* | AB201818 | Kobayashi et al., 2005 |
| RABV | Brazil: Sao Paulo State | 2000 | Nucleoprotein | *Desmodus rotundus* | AB201803 | Kobayashi et al., 2005 |
| RABV | Brazil: Sao Paulo State | 2000 | Nucleoprotein | *Desmodus rotundus* | AB201804 | Kobayashi et al., 2005 |
| RABV | Brazil: Sao Paulo State | 1998 | Nucleoprotein | *Artibeus Literatus* | AB117969 | Kobayashi et al., 2005 |
| RABV | Brazil: Sao Paulo State | 1998 | Nucleoprotein | *Artibeus Literatus* | AB117970 | Kobayashi et al., 2005 |
| RABV | Brazil: Sao Paulo State | 1998 | Nucleoprotein | *Artibeus Literatus* | AB117971 | Kobayashi et al., 2005 |
| RABV | Brazil: Sao Paulo State | 1998 | Nucleoprotein | *Artibeus Planirostris* | AB117972 | Kobayashi et al., 2005 |
| RABV | Brazil: Sao Paulo State | 1998 | Nucleoprotein | *Desmodus rotundus* | AB083807 | Ito et al, 2003 |
| RABV | Brazil: Sao Paulo State | 2002 | Nucleoprotein | *Artibeus Literatus* | AB201802 | Kobayashi et al., 2005 |
| RABV | Brazil: Sao Paulo State | 1998 | Nucleoprotein | *Nyctinomops Laticaudatus* | AB201806 | Kobayashi et al., 2005 |
| RABV | Brazil: Sao Paulo State | 1999 | Nucleoprotein | *Nyctinomops Laticaudatus* | AB201807 | Kobayashi et al., 2005 |
| RABV | Brazil: Sao Paulo State | 2001 | Nucleoprotein | *Nyctinomops Laticaudatus* | AB201808 | Kobayashi et al., 2005 |
| RABV | Brazil: Sao Paulo State | 1998 | Nucleoprotein | *Eumops Auripendulus* | AB201809 | Kobayashi et al., 2005 |
| RABV | Brazil: Sao Paulo State | 2001 | Nucleoprotein | *Eptesicus Furinalis* | AB201812 | Kobayashi et al., 2005 |
| RABV | Brazil: Sao Paulo State | 2001 | Nucleoprotein | *Eptesicus Furinalis* | AB201813 | Kobayashi et al., 2005 |
| RABV | Brazil: Sao Paulo State | 2002 | Nucleoprotein | *Eptesicus Furinalis* | AB201814 | Kobayashi et al., 2005 |
| RABV | Brazil: Sao Paulo State | 1999 | Nucleoprotein | *Molossus molossus* | AB201815 | Kobayashi et al., 2005 |
| RABV | Brazil: Sao Paulo State | 2002 | Nucleoprotein | *Molossus molossus* | AB201816 | Kobayashi et al., 2005 |
| RABV | Canada: Saskatchewan | 1989 | Nucleoprotein | *Eptesicus fuscus* | AF351932 | Nadin-Davis et al., 2001 |
| SHIBV | Kenya | 2009 | Complete | *Hipposideros commersoni* | GU170201 | Kuzmin et al. 2010 |
| WCBV | Russia: Krasnodar | 2002 | Complete | *Miniopterus schreibersi* | EF614258 | Kuzmin et al. 2005 |
