# Supplementary figures and images for: The Global Phylogeography of Lyssaviruses - Challenging the 'Out of Africa' Hypothesis
Source: PLoS Negl Trop Dis. 2016 Dec 30;10(12):e0005266. doi: 10.1371/journal.pntd.0005266 (PMC5231386; doi:10.1371/journal.pntd.0005266)

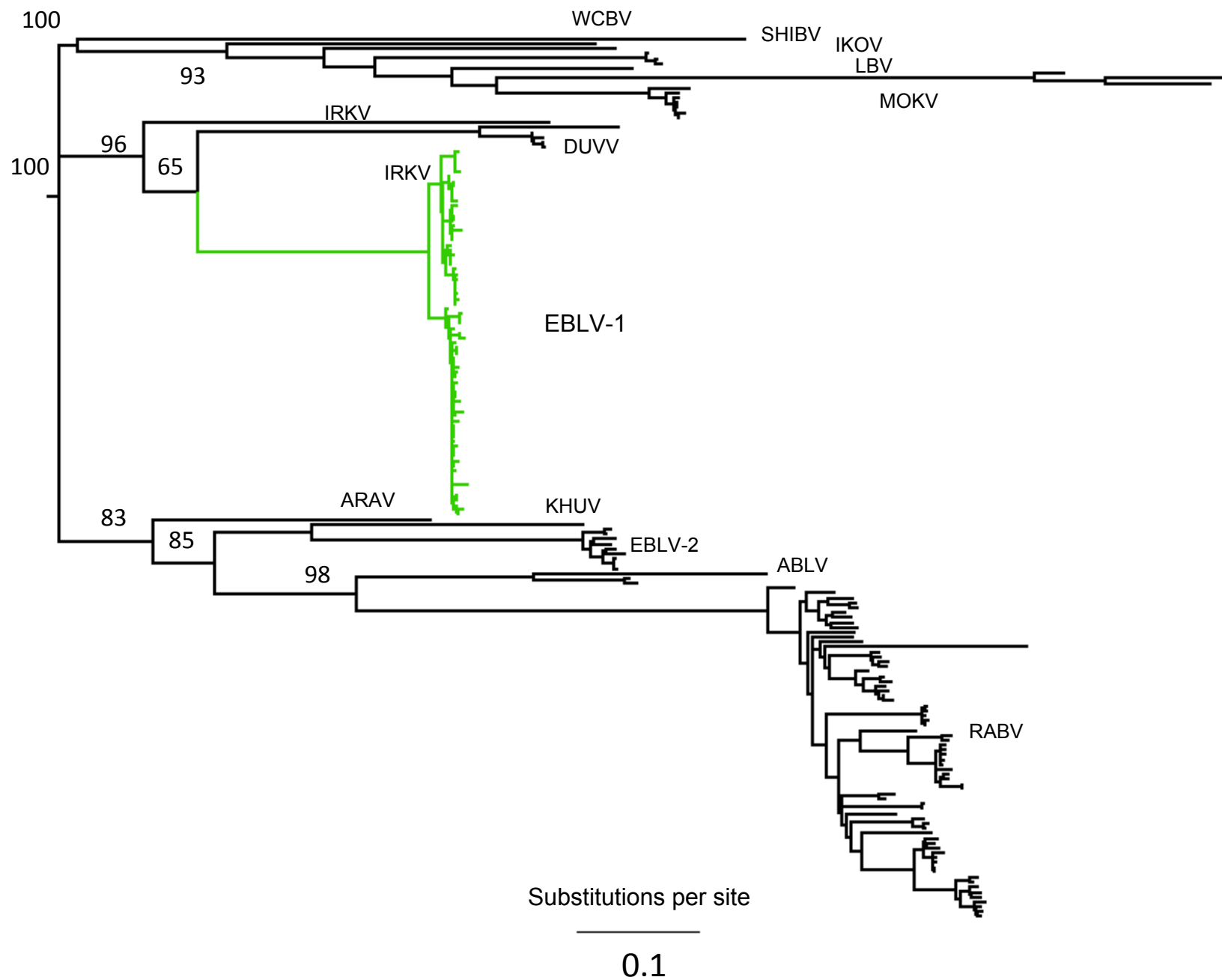

Supplement: S1 Fig — (PDF) [file pntd.0005266.s002.pdf]

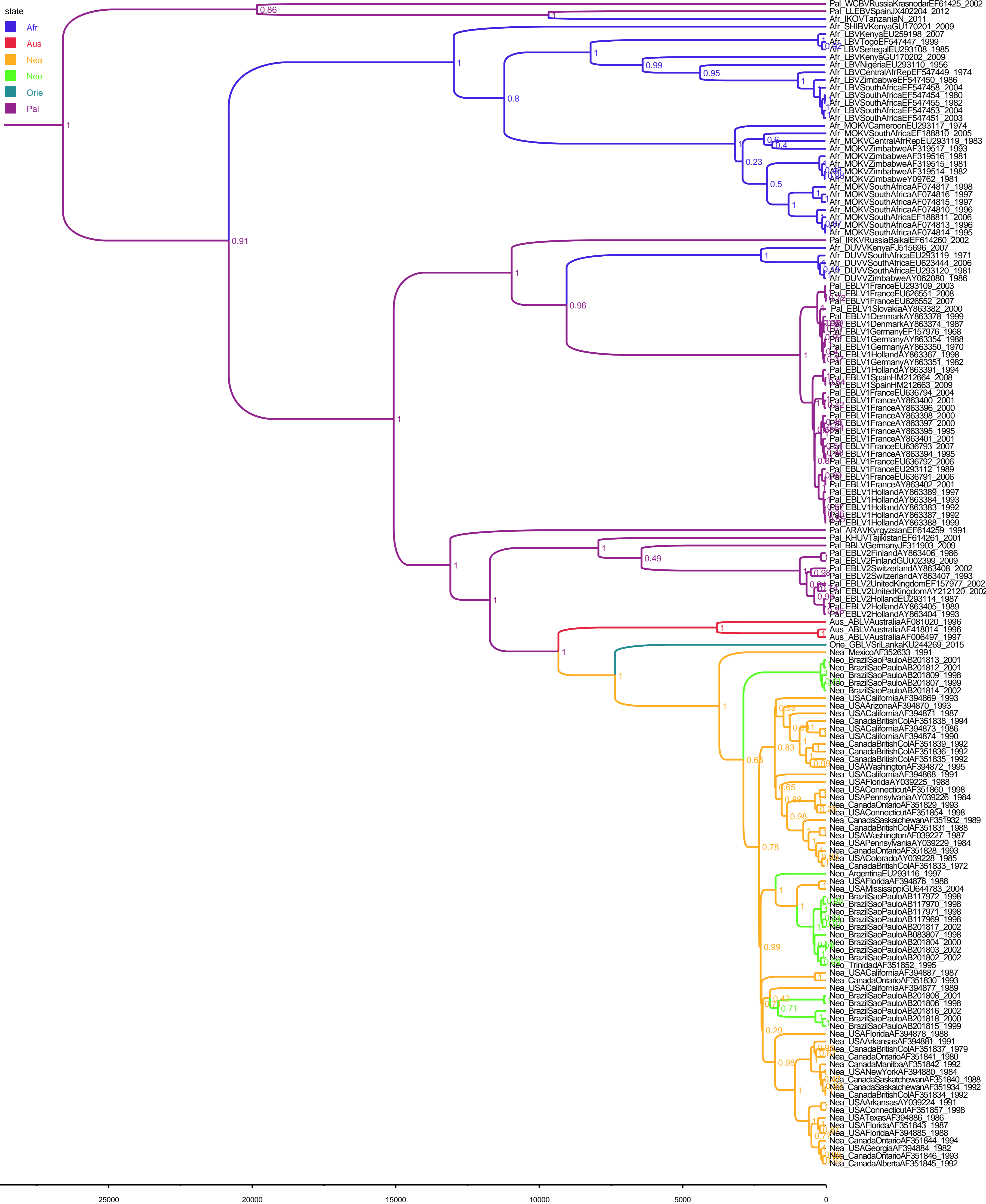

Supplement: S2 Fig — Support values corresponding to Bayesian posterior probabilities are indicated. Tips are labelled with the following information: Ecozone, follow by the_species name, country where it was isolated, GenBank accession number and year of isolation. Virus names and other details are as Fig 1. (PDF) [file pntd.0005266.s003.pdf]

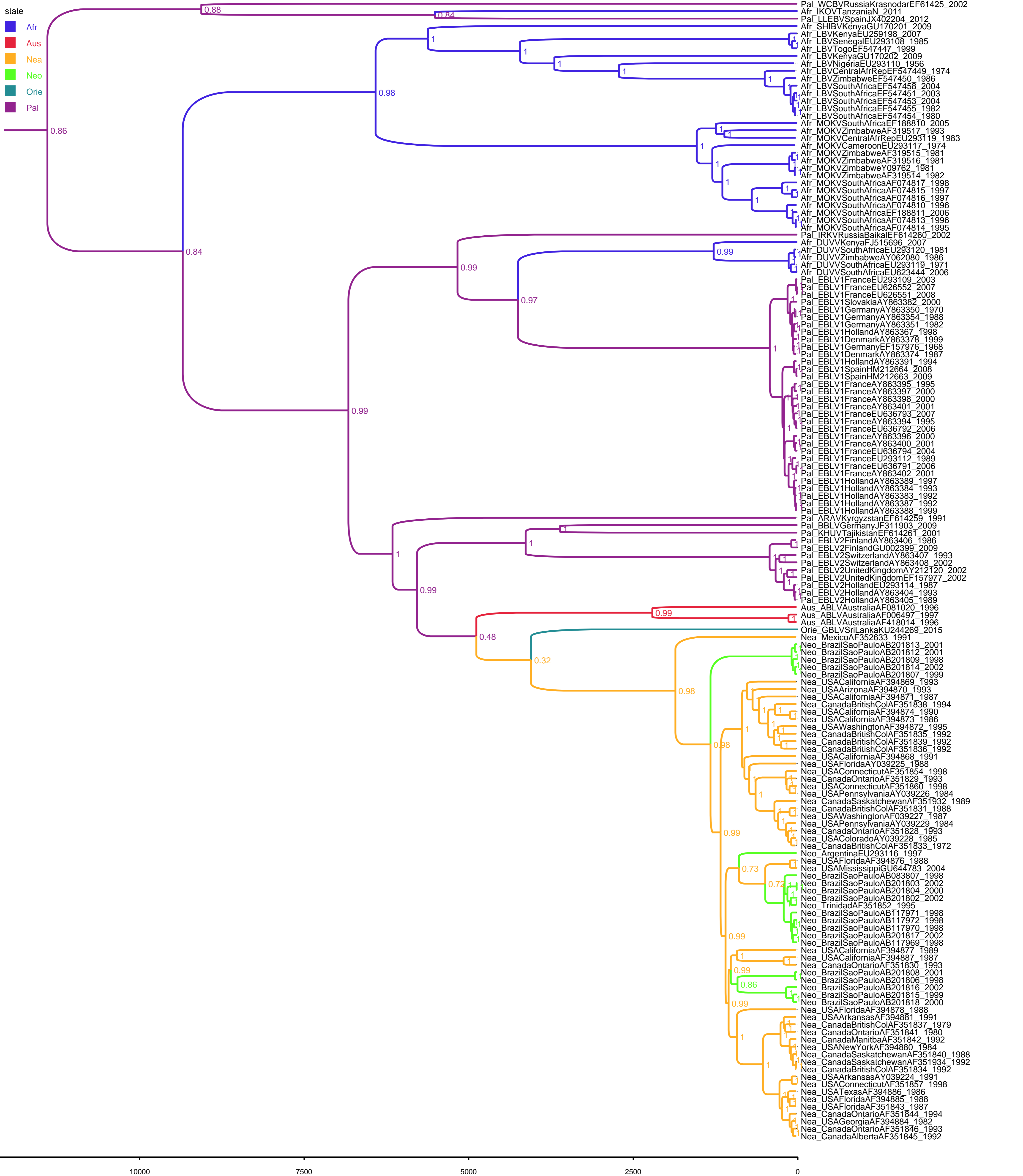

Supplement: S3 Fig — The phylogenetic tree was generated from 153 nucleoprotein gene sequences and inferred with a Lognormal relaxed-clock Bayesian analysis using BEAST. Support values corresponding to Bayesian state probabilities from the different assigned ecozones are indicated. Tips are labelled with the following information: Ecozone, follow by the species name, country where it was isolated, GenBank accession number and year of isolation. Virus names and other details are as Fig 1. (PDF) [file pntd.0005266.s004.pdf]

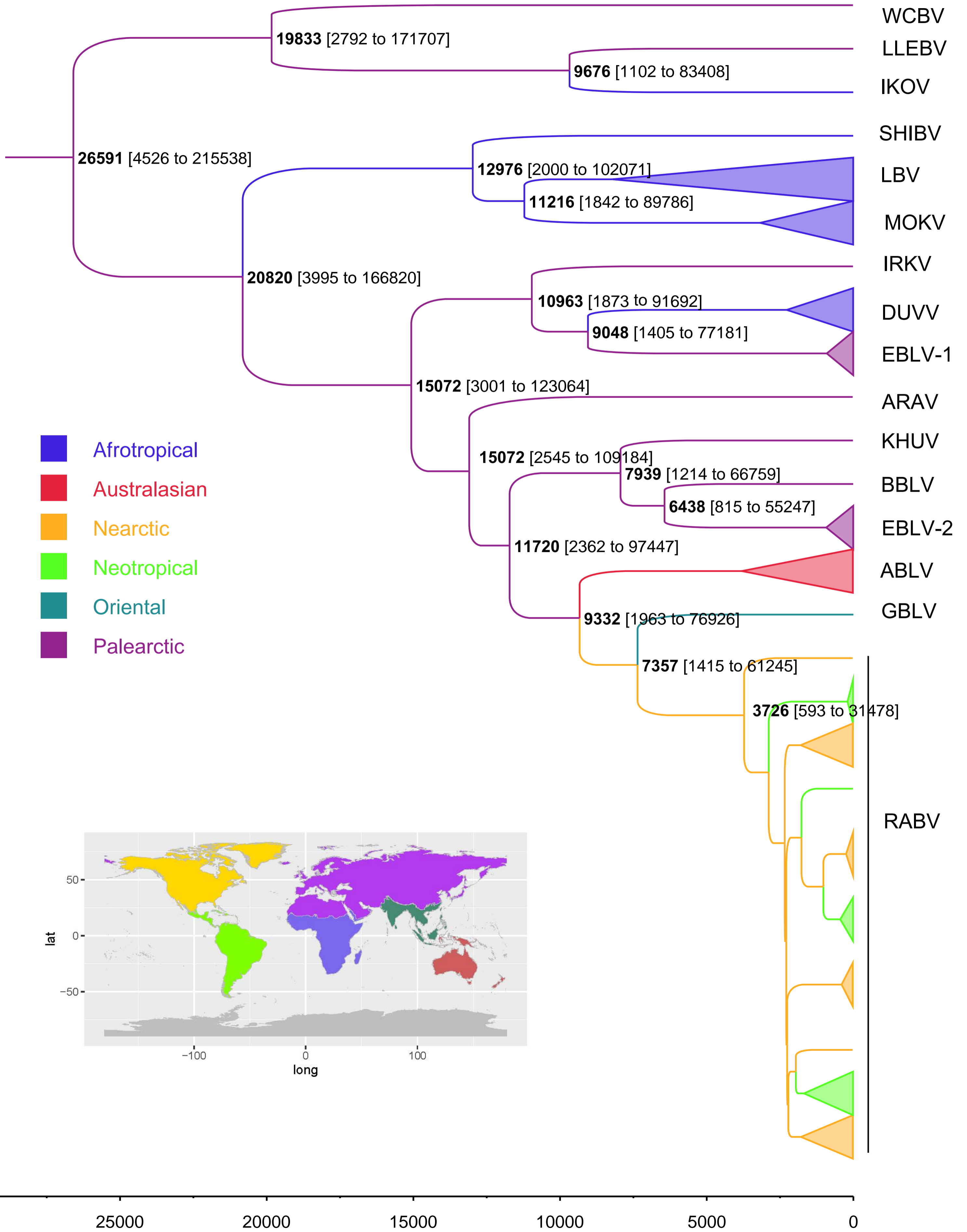

Supplement: S4 Fig — The phylogenetic tree was generated from 153 nucleoprotein gene sequences and inferred with a Lognormal relaxed-clock Bayesian analysis using BEAST showing divergence times in years with 95% credible intervals. Branch colours correspond to ecozones shown on the map. The time scale is in years. Virus names are as Fig 1. (PDF) [file pntd.0005266.s005.pdf]
