# Supplementary material for: The Global Phylogeography of Lyssaviruses - Challenging the 'Out of Africa' Hypothesis
Source: PLoS Negl Trop Dis. 2016 Dec 30;10(12):e0005266. doi: 10.1371/journal.pntd.0005266 (PMC5231386; doi:10.1371/journal.pntd.0005266)
Supplement: S5 Fig — Coloured pie-charts represent proportions generated from the different assigned states of the character (see colour legends). The grey terminal pie-chart indicated a polymorphic state that was coded as uncertain in the data matrix for RABV species. Support values are indicated above branches and correspond to bootstrap and posterior probabilities, respectively. Virus names are as Fig 1. (PDF) [file pntd.0005266.s006.pdf]

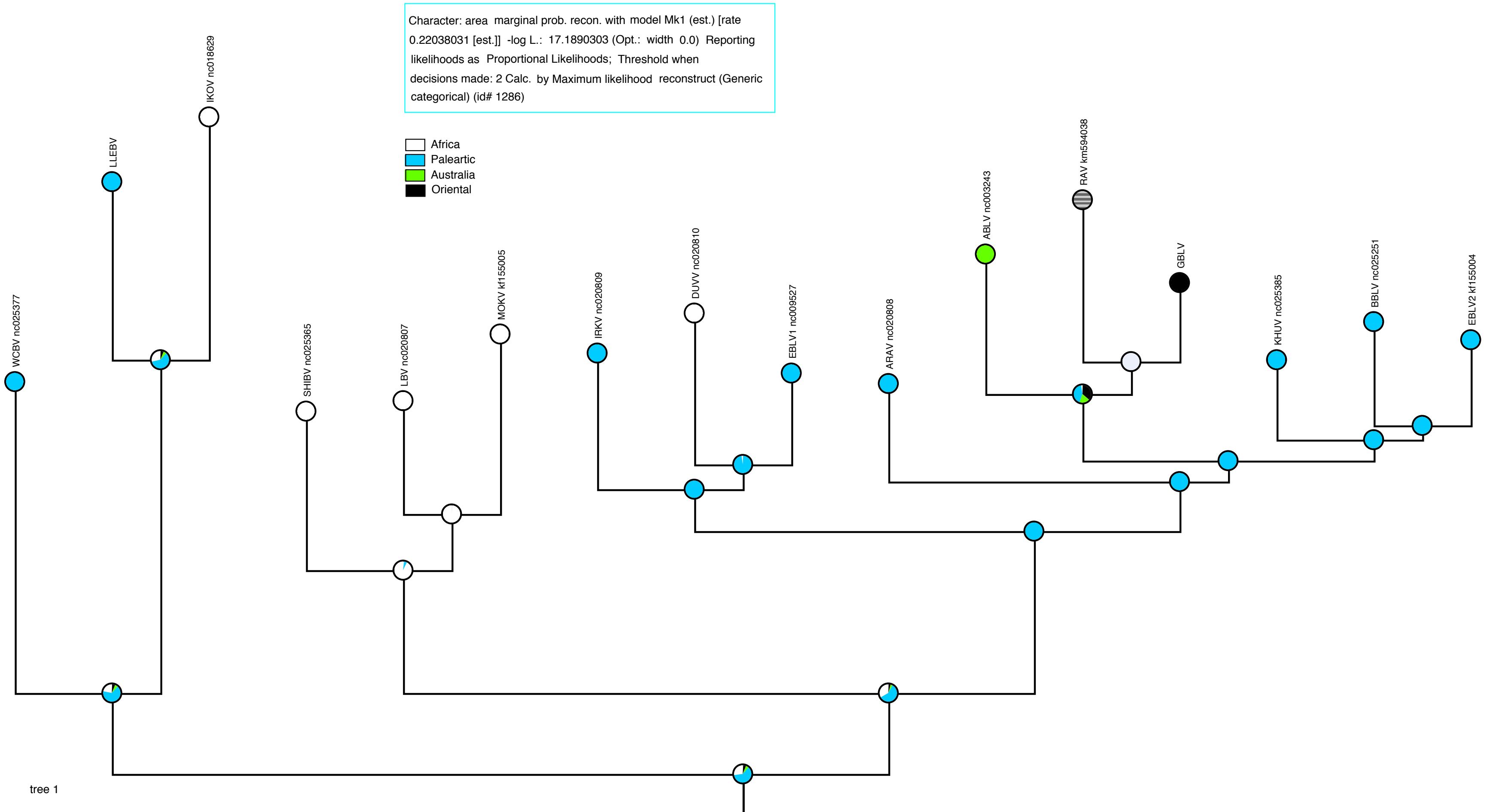

Character: area marginal prob. recon. with model Mk1 (est.) [rate 0.22038031 [est.]] -log L.: 17.1890303 (Opt.: width 0.0) Reporting likelihoods as Proportional Likelihoods; Threshold when decisions made: 2 Calc. by Maximum likelihood reconstruct (Generic categorical) (id# 1286)
Africa
Paleartic
Australia
Oriental
WCBV nc025377
LLEBV
IKOV nc018629
SHIBV nc025365
LBV nc020807
MOKV kf155005
IRKV nc020809
DUVV nc020810
EBLV1 nc009527
ARAV nc020808
ABLV nc003243
RAV km594038
GBLV
KHUV nc025385
BBLV nc025251
EBLV2 kf155004
tree 1
